# Multi-method process maps: An interdisciplinary approach to investigate *ad hoc* modifications in protocol-driven interventions

**DOI:** 10.1017/cts.2020.14

**Published:** 2020-02-26

**Authors:** Thomas I. Mackie, Leah Ramella, Ana J. Schaefer, Monica Sridhar, Alice S. Carter, Abbey Eisenhower, Grace T. Ibitamuno, Marisa Petruccelli, Shawna V. Hudson, R. Christopher Sheldrick

**Affiliations:** 1 Rutgers School of Public Health, Piscataway, NJ, USA; 2 Institute for Health, Health Care Policy and Aging Research, Rutgers University, New Brunswick, NJ, USA; 3 Department of Health Law, Policy and Management, Boston University School of Public Health, Boston, MA, USA; 4 Rutgers Robert Wood Johnson Medical School, New Brunswick, NJ, USA; 5 Department of Clinical Psychology, University of Massachusetts, Boston, MA, USA; 6 Department of Family Medicine, UMDNJ-Robert Wood Johnson Medical School, New Brunswick, NJ, USA

**Keywords:** Implementation science, modification, adaptation, process assessment

## Abstract

**Introduction:**

Implementation scientists increasingly recognize that the process of implementation is dynamic, leading to *ad hoc* modifications that may challenge fidelity in protocol-driven interventions. However, limited attention to *ad hoc* modifications impairs investigators’ ability to develop evidence-based hypotheses about how such modifications may impact intervention effectiveness and cost. We propose a multi-method process map methodology to facilitate the systematic data collection necessary to characterize *ad hoc* modifications that may impact primary intervention outcomes.

**Methods:**

We employ process maps (drawn from systems science), as well as focus groups and semi-structured interviews (drawn from social sciences) to investigate *ad hoc* modifications. Focus groups are conducted with the protocol’s developers and/or planners (the implementation team) to characterize the protocol “as envisioned,” while interviews conducted with frontline administrators characterize the process “as realized in practice.” Process maps with both samples are used to identify when modifications occurred across a protocol-driven intervention. A case study investigating a multistage screening protocol for autism spectrum disorders (ASD) is presented to illustrate application and utility of the multi-method process maps.

**Results:**

In this case study, frontline administrators reported *ad hoc* modifications that potentially influenced the primary study outcome (e.g., time to ASD diagnosis). *Ad hoc* modifications occurred to accommodate (1) whether providers and/or parents were concerned about ASD, (2) perceptions of parental readiness to discuss ASD, and (3) perceptions of family service delivery needs and priorities.

**Conclusion:**

Investigation of *ad hoc* modifications on primary outcomes offers new opportunities to develop empirically based adaptive interventions. Routine reporting standards are critical to provide full transparency when studying *ad hoc* modifications.

## Introduction

Implementation science seeks to improve the effectiveness and quality of healthcare across diverse contexts by facilitating systematic uptake of evidence-based practices [1]. Across several frameworks [2,3], authors have identified over 23 factors influential to implementation at the personal, organizational, or community levels [4]. These factors can result in modifications to protocol-driven interventions in unanticipated ways (hereafter, “*ad hoc* modifications” [5]) and ultimately impact the associated outcomes.

Despite increased recognition of the potential influences *ad hoc* modifications have on primary outcomes [6–9], there remain limited tools available to systematically identify modification made to protocol-driven interventions during implementation. While the need for clear cataloguing of interventions “as envisioned” is increasingly recognized (e.g., TIDieR checklist and guide as completed by the implementation team) [10,11], relatively few methods are available to investigate *ad hoc* modifications [12]. Integration of methods from the system and social sciences offers unique opportunities to facilitate systematic collection of data necessary to investigate *ad hoc* modifications made by frontline administrators in protocol-driven interventions. We propose a multi-method approach both to identify *ad hoc* modifications hypothesized to be influential to outcomes (i.e., effectiveness and/or cost) and to the development of future implementation strategies. Specifically, we propose an interdisciplinary multi-method approach employing process maps, focus groups, and semi-structured interviews. We then illustrate its use by detailing a case example.

### Defining *Ad hoc* Modifications and Implementation Fidelity

Challenges to protocol adherence are increasingly recognized. Modification, as defined by Stirman et al., is any change made to the interventions, whether a purposeful alteration, as in the case of planned adaptation, or changes made in response to unanticipated challenges [8]. Notably, this definition flexibly includes changes to protocol-driven interventions that are unplanned and/or unanticipated by the developer/researcher [13]. In this paper, we differentiate between two types of modifications. On the one hand, we define “planned adaptations” as modifications that are deliberately pursued for a given purpose, such as to facilitate fit and/or improve effectiveness in a given context [14]. In contrast, other modifications may be implemented that are reactive, unplanned, and may have been unanticipated by the developers/researchers; we define these as “*ad hoc* modifications” [15].

### Justification for Novel Methods to Study *Ad hoc* Modifications in Protocol-driven Interventions

The study of *ad hoc* modifications facilitates investigation of intervention fidelity or the “the degree to which an intervention happened in the way the investigators intended it to” [16]. Given the translational challenges in replicating protocol-driven interventions in community-based settings, traditional paradigms of unidirectional movement from evidence to practice are increasingly tempered by understandings of implementation as a bidirectional exchange of knowledge, values, and beliefs (referred to as “cultural exchange”) between the developers and frontline administrators [17,18]. Our focus on *ad hoc* modifications highlights both the different “world view” of frontline providers, the discretion provided to those who implement protocols, and the potential for unanticipated modifications to intervention protocols. Frontline administrators in community-based settings generally seek to serve a population rather than to test a theoretical model and thus may modify programs to address the perceived needs [19] or characteristics of their clients [20–23] and a host of other contextual factors (e.g., finances, agency capacities, funding requirements, or political climates) [24,25]. Implementation science studies increasingly recognize frontline administrators may deviate from an intervention protocol when knowledge exchange and alignment of values are inadequate [17,18]. While this literature draws attention to why *ad hoc* modifications occur within protocol-driven interventions [26,27], less attention has been given to the influences of these modifications on key implementation and intervention outcomes.

*Ad hoc* modifications may occur at various stages of the implementation process. By systematically investigating *ad hoc* modifications in protocol-driven interventions, new opportunities exist to identify the extent and impact of both anticipated and unanticipated modifications on key outcomes [15]. Typically, implementation studies focus on anticipated modifications by employing *a priori* constructs of implementation fidelity [28,29]. In contrast, *ad hoc* modifications may include but are not limited to constructs typically included in fidelity assessment protocols. Moreover, they may have very different effects, with the ability to either enhance or degrade the (1) integrity of core intervention elements, (2) core implementation strategies, and/or (3) primary outcomes associated with implementation [30].

In this paper, we propose interdisciplinary multi-method process maps to facilitate the systematic investigation of *ad hoc* modifications for protocol-driven interventions. Consistent with the Framework for Reporting Adaptations and Modifications-Enhanced (FRAME) [12], our approach seeks to generate evidence-based hypotheses on how adaptation might impact effectiveness and cost [13]. Multi-method process maps aims to provide information needed for later delineation by the researchers, developers, and/or implementation team to determine whether or not the modifications identified are fidelity-consistent, defined “as those which do not alter core elements of treatment significantly enough to reduce adherence to a protocol and do not reduce ability to differentiate between treatments” [14]. Bridging traditions from system and social sciences, we propose and illustrate application of a systematic multi-method approach employing process maps, semi-structured interviews, and focus groups to improve early detection of autism spectrum disorder (ASD) in community-based early intervention (EI) agencies.

## Methods

### Overview of Methods

Process mapping offers one approach for examining implementation and is commonly used in healthcare settings for quality improvement to (1) determine actions required to deliver an intervention, (2) document types of actions required and order of actions taken, and (3) define boundaries between actions required for implementation and actions with other purposes [31]. Process maps can also reveal system variation and identify opportunities for performance optimization [32,33]. Process maps frequently provide data for other types of analyses, such as time-use studies to inform time-driven activity-based costing [34].

Our multi-method process maps draw on rigorous standards for qualitative research, including both respondent and methodological triangulation. First, a third-party and independent evaluation team engages the team who developed and planned implementation of the intervention protocol (hereafter, “the implementation team”). The implementation team is sampled to characterize the process “as envisioned.” Second, the individuals who deliver the intervention (hereafter, “front-line administrators”) are sampled to investigate how implementation was “realized in practice.” Finally, efforts may be taken to validate findings through “member-checking” focus groups. See Table 1 for a summary of the approach.

**Table 1.** Stages of multi-method process mapping

Stage. Goal	Sample	Method	Analytic approach
**Stage 1.** To characterize “process as envisioned”	Implementation team (including any stakeholder informing the process “as envisioned”)	1. Introduce and complete process maps, individually 2. Conduct focus group to generate consensus on process “as envisioned”	*A priori* and/or emergent coding structure in a process of *coding consensus, co-occurrence, and constant comparison* to characterize: a. Planned adaptations (“as envisioned”) and *ad hoc* modifications (“as realized in practice”); b. When and how modifications occurred; and c. Content and context, and motivation.
**Stage 2.** To characterize “process as realized in practice”	Frontline administrators (including any stakeholders informing process “as realized in practice”)	Semi-structured interviews, with guided process map activity	
**Stage 3.** To validate study findings (optional).	Implementation team and/or frontline administrators	Focus groups, presenting process maps, and/or summary of qualitative tables (e.g., Tables 3, 4, and 5)	A priori and/or emergent coding structure to capture whether respondents are in agreement with synthesis of study findings and rationale for concordant/discordant perspective.

### Conceptual Model to Characterize Modifications

Prior research on modifications of evidence-based practices proposes common frameworks to characterize modifications. Specifically, Stirman et al. (2013) initially proposed characterizing (1) the decision-maker, (2) modifications to context, and (3) the types and level of modification to content [8]. First, decision-makers are defined as those who are able to make the modifications (e.g., frontline administrators, supervisors, evaluation researchers, intervention developers) [8]. Each decision-maker holds unique roles and distinct motivations and may apply unique strategies resulting in *ad hoc* modifications. These varied perspectives can be critical to investigating the presence of *ad hoc* modifications, the motivations that underlie them, and the potential impact of such modification on intervention outcomes. Second, adaptations may include changes to context (e.g., format of delivery, setting, personnel, population) [8]. Third, content modifications may vary in both level (e.g., individual, cohort, population, provider, unit/organization) and type (e.g., tweaking/refining, adding/removing elements, shortening/lengthening) [12]. Derived from an evidence review of the extant literature [8], this framework is not exhaustive but provides an organizing model that is routinely used in studies of planned adaptation [35,36]. Stirman et al.’s original framework served as a foundation for our interview guide to consider *ad hoc* modification to protocol-driven interventions more broadly. Consistent with the expanded framework published by Stirman et al. in 2019, our approach also recognized the importance of investigating other dimensions of modifications, including when and how the modification were made, who decided to make the modifications, the reasons for and factors influential to the modifications, and whether planned or *ad hoc* [12].

### Conducting Focus Groups of Implementation Team Members

The implementation team includes all team members who inform the work “as envisioned,” including any planned adaptations that may depart from prior practice. Therefore, participants ideally include the developers of the protocol [PIs and Co-Is], as well as those involved in supporting protocol implementation [Research Assistants]. The implementation team also optimally includes all engaged in development of the protocol-driven intervention “as envisioned,” including supervisors or frontline administrators at the agencies when protocols are established in collaboration.

All implementation team members are assembled to introduce and distribute process map handouts. To anchor respondents in their own perspective prior to group engagement, participants initially complete process maps independently. The worksheet requires respondents to: (1) establish boundaries for what was and was not included in the intervention protocol, (2) list and order actions taken, (3) assign appropriate symbols to classify each action, and (4) add arrows to indicate direction flow.

Focus groups are subsequently conducted to reveal variation in perspectives across team members and to generate consensus on implementation “as envisioned.” Notably, process mapping typically includes more detail than schematics or logic diagrams that may be readily available from grant applications or IRB protocols. We recommend identifying one person to diagram the process map for the group initially to expedite the process.

Subsequently, a focus group guide inquires on: (1) any differences in the start/end points of the intervention process, (2) types of actions used to deliver the intervention steps and decision points, and (3) the order of actions taken. The trained facilitator inquires about dimensions of planned adaptation based on Stirman’s et al. 2013 framework, including the (1) decision-maker, (2) the context, and the (3) level and type of content [8]. Facilitator seeks consensus in describing the process “as envisioned.” If consensus is not reached and variations exist, this should also be represented in the process map.

### Conducting Semi-Structured Interviews of Frontline Administrators

Participants include any decision-makers (e.g., frontline administrators, patients, supervisors, and other frontline decision-makers) relevant to generating *ad hoc* modifications of a given protocol-driven intervention. Data collection should occur until data are sufficient to answer the posed research question in accordance with the selected qualitative standard (e.g., thematic saturation) [37]. For example, *ad hoc* modifications may be hypothesized to vary across sites; in these cases, thematic saturation would optimally be achieved among staff at each site.

Individual interviews include a process map activity and are conducted with frontline administrators to provide an in-depth understanding and global assessment of implementation “as realized in practice.” We employed individual interviews with frontline administrators to examine *ad hoc* modifications at the provider level. Planned adaptations by the implementation team, in contrast, benefit from a consensus-driven process given the need for identification of a process “as envisioned.”

The process map activity is facilitated by the interviewer asking questions to: (1) establish the start/end point of the protocol, (2) identify types of actions used to deliver the intervention (steps, decision points, and wait times), (3) list and order actions taken, (4) assign appropriate symbols to classify each action, and (5) add arrows to indicate directional flow. Questions also focus on dimensions of *ad hoc* modifications, including (1) the decision-maker, (2) the context of the modification (format, setting, population, and personnel), (3) the level (individual recipient, population, cohort, facilitator, unit-level, etc.), and (4) the type of content. After completion, the interviewer reviews the process map to identify potential areas of variation and confirm accurate representation.

### Qualitative Analysis

For multi-method process maps, qualitative analyses employ *a priori* and/or emergent coding structure to characterize *ad hoc* modifications and motivations, organized by the specific action taken, and the motivations for those actions. *A priori* codes optimally employ an existing framework (e.g., Stirman et al.’s FRAME). The codebook is systematically applied through a from analytic approach (e.g., “coding consensus, co-occurrence, and constant comparison” [38]). Comparative analyses are conducted to identify *ad hoc* modifications within and across programs. Multi-method process maps aim to generate discrete hypotheses regarding *ad hoc* modifications that may increase or decrease primary outcomes. However, a direct evaluation of impact is beyond the scope of this method. “Member-checking” focus groups with implementation team and/or frontline administrators may also be conducted to improve validity of the findings. An illustrative case study is provided below.

## Case Study: A Protocol-Based Intervention to Improve Early Identification of ASD among Young Children in EI

Our case example focuses on *ad hoc* modifications within a community-based research project that utilized a Type II effectiveness-implementation hybrid design to address racial/ethnic disparities in time to ASD diagnosis and access to services [39]. The protocol-driven intervention consisted of two stages of screening and a subsequent diagnostic assessment. The Stage 1 questionnaire and the Stage 2 observational screenings were embedded into the routine clinical care provided by EI providers (i.e., frontline administrators), while implementation team members conducted a university-based diagnostic assessment for ASD. The complete description of the protocol-driven intervention is available in prior publications [19].

The present study primarily sought to identify whether and how frontline administrators modified the protocol-driven intervention to influence the primary outcome, time to diagnosis. Methods and results are presented in three parts: (1) assessment of work “as envisioned,” (2) assessment of work “as realized in practice,” and (3) comparison of work as “envisioned” to process “as realized in practice.” Ethical review was provided by the Institutional Review Board at University of Massachusetts Boston; informed consent was obtained from all study participants.

### Focus Groups with Implementation Team: Protocol “as Envisioned”

#### Sample

We recruited the implementation team, including the Principal Investigators and Co-Investigators of the study (*n* = 4) and research staff overseeing day-to-day operations within the three sites (*n* = 5). Our sampling approach sought to facilitate expertise on the intervention protocol, itself, as well as the planned adaptations that occurred in agency-level implementation.

#### Process map procedures

All participants were initially assembled and introduced to what process maps are and how to complete them; they were then provided a handout with written directions and a process map template for completion. Implementation team members were asked to document the process that they knew best, whether that referred to the original intervention protocol or an agency-specific protocol.

#### Focus groups procedures

After individual process maps were generated, focus groups facilitated a characterization of the process and to reconcile and create consensus on the protocol and planned adaptations. Due to time limitations, one implementation team member represented her process map and then characterized differences and site-specific adaptations. An independent evaluation team then asked the implementation team the following questions, “Does this match your characterization of [this stage of the process]?” “Would you want to change anything?” “Do you want to add anything?” and then asked to explain any position provided. A process of consensus was used and achieved in characterizing the process and representing the planned adaptations made across agencies.

#### Focus group analysis

**Fig. 1.**
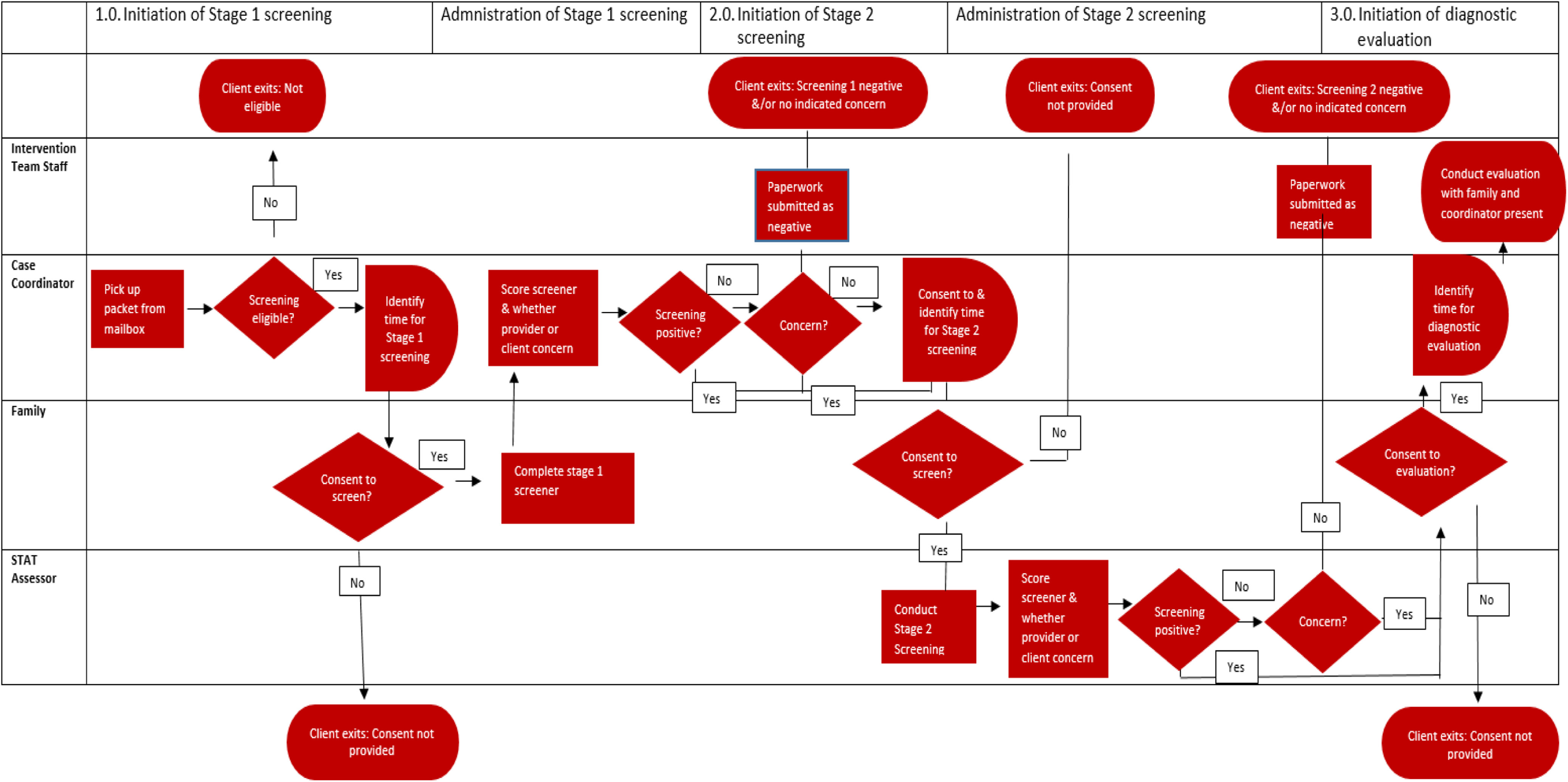
Process map of multistage screening protocol “as envisioned.”

Key elements of the process were documented in the collectively generated process map with clarifying questions provided as needed. The final model was constructed in a swimming lane diagram to identify the individuals conducting each action. During the consensus process, planned adaptations were identified and then modeled using Microsoft Visio (2000). To facilitate readability, we simplified the process map in Fig. 1.

### Semi-Structured Interviews with EI Providers: Protocol “as Realized in Practice”

#### Interview sample

Our study purposively sampled the EI providers who administered the multistage screening protocol and were central decision-makers in making *ad hoc* modifications. Providers were asked to describe the type of and motivation for *ad hoc* modifications. We hypothesized potential agency variation in *ad hoc* modifications made by providers so sought theoretic saturation across and within each agency [37]. Sampling concluded when no new data appeared and the types and motivations for variation were well developed within sites and across providers. Table 2 provides sociodemographic characteristics of our sample.

**Table 2.** Characteristics of front-line administrators (*n* = 52)

Age, years (range)	34.3 (25–62)
Gender, *n* (%)	
F	52 (100)
M	–
Race, *n* (%)	
Non-Hispanic White	40 (77)
Hispanic Latino (not Hispanic Black)	6 (12)
Asian	3 (6)
Non-Hispanic Black	1 (1)
Multiracial	2 (4)
Education level, *n* (%)	
BA/BS	18 (35)
MA	34 (65)
Participants by early intervention agency, *n* (%)	
Site 1	21 (40)
Site 2	14 (27)
Site 3	17 (33)
Specialized credentialing/training, *n* (%)*	
Education	12 (23)
Special education	6 (12)
Speech/language	8 (15)
Music therapy	2 (4)
Occupational therapy	7 (13)
Trained in Stage 2 screening tool, *n* (%)	
Yes	26 (50)
No	21 (40)
Not reported	5 (10)
Language spoken	
English only	29 (56)
English and Spanish	17 (32)
Spanish only	3 (6)
English and other	3 (6)
Tenure in position	5.2 (3 months–23 years)

* Providers of the protocol-based interventions held multiple credentials totaling percentages of credentialing to greater than 100% of the sample.

#### Process map, interview procedures, and measures

Interviews, with an embedded process map activity, were conducted individually in-person to provide an in-depth understanding of implementation “as realized in practice.” Interviews took approximately 1 hour to complete and were conducted by a trained member of the research team. The interview guide employed the measures described in the overview of the method; specific interview guide questions used are available in the supplement. An illustrative example of the process map “as realized” in practice is also available in supplemental materials.

#### Data analysis

To analyze qualitative data from semi-structured interviews, we employed a modification of grounded theory referred to as “Coding Consensus, Co-occurrence, and Comparison” [40]. Analyses are derived from the data and then illustrated by characteristic examples of the data. The codebook was developed by an interdisciplinary team of researchers with expertise in medical sociology, health services research, clinical psychology, and medicine. The team initially coded transcripts independently at a general level to condense the data into analyzable units. These researchers assigned codes to segments of text ranging from a phrase to several paragraphs based on emergent themes (i.e., modifications); data were analyzed by multistage screening phase so that the codebook aligned with the sequential description of process facilitated by the process map activity. The researchers then coded each text and met to facilitate consensus. When disagreements in assignment or description emerged, researchers resolved these through discussion, thereby enhancing the codebook. Through this process of consensus among team members, the final list of codes consisted of a numbered list of modifications organized by the stage of the screening process as “realized in practice.” Analysts subsequently conducted open-ended coding of the identified modifications to identify the motivation of the modification, the content of the protocol modification, and potential impact on screening protocol. Results were summarized in a table indicating an illustrative quote and the total frequency of participants indicating each theme by site.

### Results

#### Comparison of protocol as “envisioned” to process “as realized in practice”

Comparison of process maps created by the implementation team and EI providers (as frontline administrators) facilitated characterization of modifications during each subsequent stage of the process, specifically: (1) the initiation and implementation of Stage 1 screening, (2) Stage 2 screening, and then (3) the ASD diagnostic assessment.

The implementation team indicated that initiation of the Stage 1 screening tool occurs when a packet of study materials (including consent and screening tools) arrived to the provider’s mailbox. Administrators reported *ad hoc* modifications of the protocol-driven intervention that would speed or lengthen the process “as envisioned,” for example, by requesting receipt of a packet before it was provided or by delaying administration after receipt. As depicted in Fig. 1, the protocol “as envisioned” requires parental consent in order to advance to each of the three stages throughout the screening protocol – an element that was not present in simplified descriptions of the process included in the grant protocols. Additional decision criteria included review of eligibility criteria for EI services and the potential for children to “age out” of services and therefore eligibility to participate in the screening protocol. Wait periods were only present at Stages 2 and 3 to coordinate scheduling with the screening team. Results are provided in Fig. 1. Results of findings are positioned in relation to the specific modifications reported by providers between the process “as envisioned” and “as realized” in practice below.

Table 3 characterizes several *ad hoc* modifications to Stage 1 screening and reports the multiple reasons articulated by respondents for making them, including (1) the age of the child, (2) assessment of parental concern and readiness, (3) the provider’s own clinical concerns, (4) provider’s competing tasks, (5) prioritization of other child and family needs, (6) established rapport with the family, and (7) the location of where the parent receives services (e.g., home versus childcare). Across the three sites, providers’ perceptions of family readiness/parental concern and provider concern most frequently motivated these *ad hoc* modifications.

**Table 3.** *Ad hoc* modifications that lengthen/extend/shorten initiation and implementation of Stage 1 screening

	Motivation for modification	Content	Illustrative quote	Participants by site		
				Site 1	Site 2	Site 3
1.0	Stage 1 screening					
1.1	Age of child	Initiated protocol early (prior to receipt of packet) or delayed initiation (after receipt of packet).	Int: What determines when [you’ll delay providing the packet]? Resp:…it’s the age at the time you eval them.	3	1	1
1.2	Assess family readiness or parental concern	Initiated protocol early or delayed initiation.	Sometime [initiation of BITSEA/POSI] is determined by parent readiness…that’s something that I feel like is really more like, clinician finesse, or kind of like your own clinical judgment call.	7	1	9
1.3	Clarity and extent of provider concern	Initiated protocol early or delayed initiation.	…for me, if I start working with a family that I’ve never met before and I have some red flags or some concerns about anything, I immediately am like, hey let’s do this questionnaire, why don’t you just fill this out and see what it says and then when you can go from the data from there.	7	5	11
1.4	Providers’ competing tasks	Delayed initiation.	…there’s just honestly so many other things going that yeah I find it not as easy to start (the screening.)	3	0	0
1.5	Prioritization other needs for the child and family	Delayed initiation.	If I feel like there’s other things that feel more pressing, especially medical thing…or a lot of other appointment, I tend not to even mention that we have the BITSEA POSI until some of those other things get addressed.	0	2	0
1.6	Established rapport with family	Delayed initiation.	…because I find the [screening tool] has questions that if I don’t feel ready asking them about…I feel awkward. I prefer to wait and to get to know the family a little bit better.	5	3	4
1.7	Location of where family receives services	Delayed initiation.	[Starting the screening process] is slower with families that I see at daycare…	4	1	0

Table 4 presents motivations for *ad hoc* modifications to the Stage 2 screening process. Families were referred to Stage 2 screening if: (1) children scored positive on either of the two BITSEA ASD indices (i.e., ASD Problem or ASD Competence) or the POSI, or (2) EI providers reported either their own or parental concern about ASD. Implementation team members envisioned planned delays based upon families’ and providers’ schedule availability. However, providers reported that in practice, additional motivations for *ad hoc* modifications of the protocol-driven intervention included (1) assessment of family readiness or parental concern, (2) provider familiarity and ability to score BITSEA/POSI, (3) whether service coordinator is also a trained Stage 2 administrator, and (4) availability of administrators for the Stage 2 screener.

**Table 4.** *Ad hoc* modifications that lengthen/extend/shorten initiation and implementation of Stage 2 screening

	Motivation for modification	Content	Illustrative quote	Participants by site		
				Site 1	Site 2	Site 3
2.0	Stage 2 screening					
2.1	Assessment of family readiness or parental concern	Lengthening/extending	Int: So, how much time do families usually need between getting the BITSEA score and STAT? Resp: Some families we can do it in a week. Some families it can 6 months. If at all, some parents never want the SAT event though were concerning scores.	9	2	2
2.2	Provider familiarity and ability to score BITSEA/POSI	Shortening	And if it is concerning…we know right away. And usually by looking at them, since I’ve a done of them, I can kind of see if they’re concerning or not, so we can talk about this, I usually talk about the STAT right then and there…would you be interested in further following this up if there is a concern?	3	0	0
2.3	Whether service coordinator is also Stage 2 administrator	Lengthening/extending	Yeah, with my families it’s a little bit different because I’m on the team so it tends to be more a little bit faster I think for people that are certified to do the STAT just because we can have our next visit be the STAT.	1	1	2
2.4	Availability of STAT administrators	Lengthening/extending	Int: if they decide to go onto the STAT, how long does that typically take? Like how long to schedule? Resp: It kind of has depended on just like availability of people that who are trained in it.”	0	3	0

Table 5 provides the *ad hoc* modifications identified at time of initiating and implementing the developmental assessment for ASD. Families were referred for a diagnostic assessment if: (1) children scored positive on the STAT or (2) EI providers reported their own or parental concern about ASD. Implementation team members envisioned planned delays based upon availability of families and providers to schedule. Providers reported that in practice, additional motivations for *ad hoc* modifications included (1) whether child spoke English as a Second Language, (2) family readiness and consent, (3) prioritization of other needs for the child and/or family, and (4) primary care provider preference and referral.

**Table 5.** Motivations for *ad hoc* modifications that lengthen/extend/shorten initiation and implementation of Stage 2 screening

	Motivation for modification	Content	Illustrative quote	Participants by site		
				Site 1	Site 2	Site 3
3.0	Stage 2 screening motivations					
3.1	Language of child	Lengthening	“I’m coming up with a language, the capacity to also do [assessment] in Vietnamese, you know, given our huge population. There are so many kids that, over the years, we’ve had sitting here waiting that we know we could have got them fast-tracked through [implementation team] if that was possible. But they’ve had to wait for months at a hospital to get the diagnoses.”	0	2	2
3.2	Family readiness and consent	Lengthening	“For this one patient I have in mind, [scheduling the diagnostic evaluation] was about a month …before the parents definitely decided on the development evaluation.”	2	0	2
3.3	Prioritization other needs for the child and family	Lengthening	“There was …one family they needed a little time to figure out how to make it work just they had stuff going in their family life.”	0	1	0
3.4	Primary care provider preference and referral	Lengthening	“Yeah, I think some-like I have one-I actually I don’t know why he’s not referring but it’s been a struggle of, like, trying to get him a developmental appointment cause the pediatrician just hasn’t referred him even though he’s really concerning. But like that’s-I don’t think that’s super common…”	0	3	0

Notably, the process “as realized” demonstrated far greater complexity than the process “as envisioned.” Process maps created by the implementation team included an average of 16 actions, while process maps created by EI providers generated an average of 28 actions. Moreover, differences in perspective were apparent between different types of participants. For example, implementation team members reported in detail the flow and anticipated timeline for the required paperwork and administration of the tool, but lacked detail regarding the reasons for intentional delays in meeting the set timeframes. In contrast, providers reported on the process of shared decision-making in a way that included explicit decision points that depended on parent response, but lacked perspectives on other aspects of the intervention protocol (e.g., how screeners were scored). Across the multistage screening protocol, provider assessment of family readiness and consent arose as a consistent cause of modifying the protocol, specifically in extending or reducing the anticipated timeline and thereby potentially impacting the primary outcome (i.e., time to diagnosis).

## Discussion

Our paper presents a systematic, interdisciplinary, multi-method approach to investigate *ad hoc* modifications in protocol-driven interventions. Specifically, our multi-method process map approach draws from system sciences (employing process maps) and the social sciences (employing qualitative methods) to facilitate systematic documentation of *ad hoc* modifications that may influence key outcomes of a given intervention. To promote transparency, we propose five areas critical to reporting studies investigating *ad hoc* modifications, with unique considerations required in articulation of (1) study design and justification, (2) sampling framework, (3) measures, (4) the analytic approach and synthesis of findings, and (5) outcome of central interest.

First, studies of *ad hoc* modification require clear specification and justification for the study design and methods employed. Multi-method process maps intend to generate an in-depth understanding of both the intervention “as envisioned” and “as realized in practice.” Notably, we employ different methodological approaches to address these two purposes. Because the intervention “as envisioned” optimally exists as a collective and single construct, we employ focus groups to unearth variation across the implementation team and to facilitate a consensus-driven process to arrive at a collective vision. In contrast, the interviews designed to address the intervention “as realized in practice” are collected and analyzed at the individual level, recognizing that *ad hoc* modifications can occur at the level of individual providers. Studies of *ad hoc* modification will benefit from such specification and justification of methods aligned with established standards in the respective disciplines.

Second, a clear and justified sampling framework is critical to studies of *ad hoc* modifications. In the present study, we purposefully sampled individuals who were critical to development (“as envisioned”) and execution (“as realized in practice”). The case example illustrates prioritization and justification of sampling of implementation team members given the specific outcome of interest to this study, time to diagnosis. Our sampling frame included participation of intervention leadership who oversaw the development of the protocol-driven intervention as well as the research assistants who brought expertise in how the planned adaptations occurred in day-to-day practice. In sampling respondents to characterize the process “as realized in practice,” specification of which level of *ad hoc* modifications is relevant and of interest is critical. For example, our purposeful sampling approach specifically sought to identify both provider- and site-level *ad hoc* modifications. Accordingly, we sampled at least 12 EI providers from each of the 3 agencies and sampled until we reached thematic saturation by site and across providers.

Third, justification for the specific measurement approach should be described and ideally aligned with conceptual frameworks and constructs available in studies of adaptation [8]. Our approach employed process maps to anchor points of adaptation within specific stages of the protocol-based intervention. Additionally, our measures for adaptation drew upon prior frameworks to characterize modifications systematically [8].

Fourth, transparency in the analytic approach employed is critical. Our framework for “*ad hoc* modifications” was articulated prior to conducting interviews and focus groups and was based on concepts consistent with existing frameworks [8]. Notably, the overarching framework allowed for systematic data collection on these dimensions and ultimately thematic saturation to be obtained. While the Stirman framework was foundational to the interview guide’s domains, our emergent coding structure also elucidated important themes specific to this protocol-driven intervention (e.g., family readiness and concern, clarity and extent of provider concern).

Finally, motivating this work is the opportunity to characterize *ad hoc* modifications that may impact a primary outcome. *Ad hoc* modifications may support the underlying causal theory (and spirit of the intervention) or they may challenge the underlying causal theory. Drawing on our case study to illustrate this point, the causal mechanism of a multistage screening protocol might rely on the assumption that screening results will be assessed by the family and clinician as accurate and they will each follow the protocol in proceeding to the subsequent diagnostic assessment for ASD within the stated timeframe. However, findings in our current and prior work suggest that clinicians and families do not always agree with the tool’s results and instead frequently place value on their own and one another’s ASD concerns independent of screening results [19,38,40]. Such a finding suggests that the screening tool, itself, functions in concert with parents’ and providers’ concerns, knowledge, and beliefs about ASD rather than in isolation, requiring attention not only to the results of the tool but also the iterative process of shared decision-making and meaning-making [19].

Several limitations to multi-method process maps are worth noting. First, the proposed approach is subject to social desirability and recall biases. To minimize social desirability bias among participants, a third-party evaluation team collected and analyzed all data. Participants were actively engaged in implementing the protocol-driven intervention to minimize recall bias when interviewed. However, the multi-method process map is especially limited when frontline administrators do not recognize modifications are being made or are challenged in recounting modifications. Opportunities also exist for analyses not to capture fully or accurately the perspective of respondents. For our illustrative case study, our team conducted “member-checking focus groups” of central themes (e.g., role of parental concern in modifying protocol) to strengthen the validity of findings [41]. Member-checking focus groups could also be used to verify that findings corroborate with the perspective of others not engaged in the interviews [41].

Evaluation of what, when, and how modifications occur is critical to implementation science given the dynamic implementation process. Stirman et al. developed the Modification and Adaptation Checklist (MAC), available as an observational and self-report coding system intended to be used in conjunction with fidelity assessment for evidence-based practices [9]. Rabin et al. adapted the 2013 FRAME model to capture modifications in alignment with the framework of RE-AIM (Reach, Effectiveness, Adoption, Implementation, and Maintenance), in an interview guide to investigate modifications in the dimensions of *Who, What, When, and Why?* [42]. While these approaches primarily target evidence-based protocols, our approach targets complex protocol-driven interventions that may not have fidelity assessments; we therefore anchor respondents in maps of the process “as envisioned” and “as realized in practice,” thus offering flexibility for various protocol-driven initiatives and for emergent and unanticipated modifications to arise. Additional research is needed to assess the relative merits of these approaches, especially given the significant burden (resource intensive and time-consuming) that might be placed on the researcher and the participant in multi-method process maps (and other approaches reliant on intensive qualitative methods like interviews/focus groups).

Our study may also be placed within the context of implementation frameworks. For example, the Quality Implementation Framework synthesized 25 frameworks deriving four implementation phases: (1) initial assessment of host setting, (2) a structure for initial implementation, (3) ongoing structure for sustained implementation, and (4) improving future applications [43]. The Quality Implementation Framework and others place particular emphasis on the critical role of developing and sustaining a shared mental model for program implementation between the researcher/developer and the administrators [43]. Despite such efforts, shared mental models cannot be assumed and may shift over time generating *ad hoc* modifications, as demonstrated in our case example. Accordingly, the proposed approach targets the fourth phase of the Quality Implementation Framework by providing an opportunity to learn from implementation experience and improve future applications [43]. Multi-method process maps are appropriate for the pilot phase of a protocol-driven intervention to identify, understand, and potentially adapt to *ad hoc* modifications at the initial stage of evidence development. However, multi-method process maps may also be used for established interventions to inform hypotheses about whether and how *ad hoc* modifications influence effectiveness and sustainability across diverse contexts.

Multi-method process maps were developed to facilitate evaluation of an implementation process in a hybrid implementation-effectiveness study design. In such cases, rapid cycle improvements are typically not conducted. However, opportunities to characterize differences between the process “as envisioned” and “as realized in practice” might be equally as useful within quality improvement initiatives. Multi-method process method may also be well suited to inclusion in the “preparation” phase of the Multiphase Optimization Strategy (MOST) framework, which is used to inform the development of randomized trials that typically include multifactorial or adaptive designs [44].

Multi-method process maps seek to generate specific hypotheses to inform quantitative analyses. As illustrated in the case study, our study found that lack of parental and/or provider concern about ASD was accommodated to introduce delays at initiation and later screening stages. Accordingly, we tested whether referrals based on concern alone were cost-effective compared to referrals based on screening results alone (with no reported concerns). Findings suggest that in the context of our screening process, reported concerns were, in fact, stronger predictors than positive screens alone in time-to-complete referrals and that referrals based on concern alone were cost-effective overall [19]. Based on these results, we intend to further optimize our screening protocol by developing implementation strategies (e.g., decision aids) to facilitate shared decision-making when there lacks alignment between the screening results, provider concern, and parent concern. We hypothesize that these strategies will assist in reducing *ad hoc* modifications that delay time to diagnosis and ultimately improve effectiveness of the protocol-driven intervention.

In summary, while the significant advances made in characterizing and evaluating planned adaptations are noteworthy [8], it is critical that we also advance the science of studying *ad hoc* modifications in protocol-driven interventions. We illustrate the opportunity to leverage interdisciplinary and team science not only to conduct multi-method process maps but also to ensure transparent reporting when undertaking the difficult task of studying *ad hoc* modifications.

## Supplementary material

For supplementary material accompanying this paper visit https://doi.org/10.1017/cts.2020.14. click here to view supplementary material
