# Supplementary material for: Multi-method process maps: An interdisciplinary approach to investigate ad hoc modifications in protocol-driven interventions
Source: J Clin Transl Sci. 2020 Feb 26;4(3):260–9. doi: 10.1017/cts.2020.14 (PMC7348036; doi:10.1017/cts.2020.14)

Multi-method process maps: An interdisciplinary approach to investigate modifications in protocol-driven interventions

Supplementary Materials

**Available Materials**	**Page Number**
Front-line Administrator Guide [Process Map Activity]	2-4
Stage 1 Process Map Illustrative Example of Process “as envisioned”	5
Stage 2 Process Map Illustrative Example of Process “as envisioned”	6

Front-line Administrator Interview Protocol [Redacted]

I. GENERAL DESCRIPTION OF PRACTICE

IA. Tell me a little about the services that you provide here at [*SITE*] and the interventions you implement.

IB. Can you tell me about the different roles that you assume in relation to different families?

III. ASD SCREENING PROCESS AND CARE-COORDINATION

I would like to draw a map that shows the full process of the two-stage screening process. To do this, I need your help in describing what the full process includes. I want the map to represent the actual processes and not the ideal. As I draw what you describe to me, please do not hesitate to correct me if I have understood something wrong. Do you have any questions about this activity?

(*Interviewer pulls out the process mapping activity worksheet to complete with study participant. Provide clarifying answers if participant has questions*.)

Interviewer Notes: Completing the process mapping activity. <br>*Start with asking question IIIA. As you draw the process map, ask questions IIIA-IIIR (when appropriate) and prompt participants to describe the next step/event.*

IIIA. From your perspective, when does the two-stage screening process start? When do you consider the two-stage screening process to be complete? (*Indicate the two terminuses on map*)

IIIB. (*Diagram the map by asking prompting questions.*)

IIIB1. Across the two-stage screening process, what specific steps are required to complete the multi-stage screening process?

IIIB2a. What, if anything, across this process may delay or speed up completion of a specific step? What, if anything delays or speeds up progression to the next step? When does this happen? *[Confirm place in the steps previously articulated and document any additional steps arising that slow down/speed up the process in process map.]*

IIB2b. *If any instances of process delaying/speeding up,* what is the cause for the process to *[speed up or slow down]*? *Probe:* What specifically leads to this change? Would this change occur for a particular child, a subset of children or all children with whom you worked? Who

decides to slow up or slow down the process? Why do you think this occurs?

IIIC. (*When the map is complete*) Let's look at the map, again, together. *Review map with participant, confirm the accuracy, and ask them to identify time range for each component in the process.*

IIIC1. As you look at the process outlined here, what are any additional challenges that you experience during these points in EI service delivery? What are any additional facilitators that you experience during these points in EI service delivery? What improvements do you think could be made to help you in your role? What improvements do you think could benefit the ASD screening process?

(Complete questions IIID-IIIE5ii for the BITSEA/POSI and STAT if identified that these tools are used in survey.)

[Additional questions omitted.]

<u>**Process Mapping Worksheet**</u>

This worksheet is to be filled out by the interviewer and document the process described the study respondent.

Key: Basic Shapes

Ovals are beginnings and endings	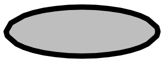	Diamonds are questions or decision points	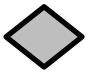
Boxes indicate procedural step taken within the processes.<br><br>o the process.	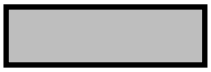	Arrows show sequence and chronologic order	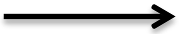
Half circle indicate a wait period having been introduced t	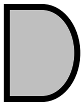		

Supplement Table 1-A. Illustrative Example of Stage 1 Screening Process Map "As Envisioned"

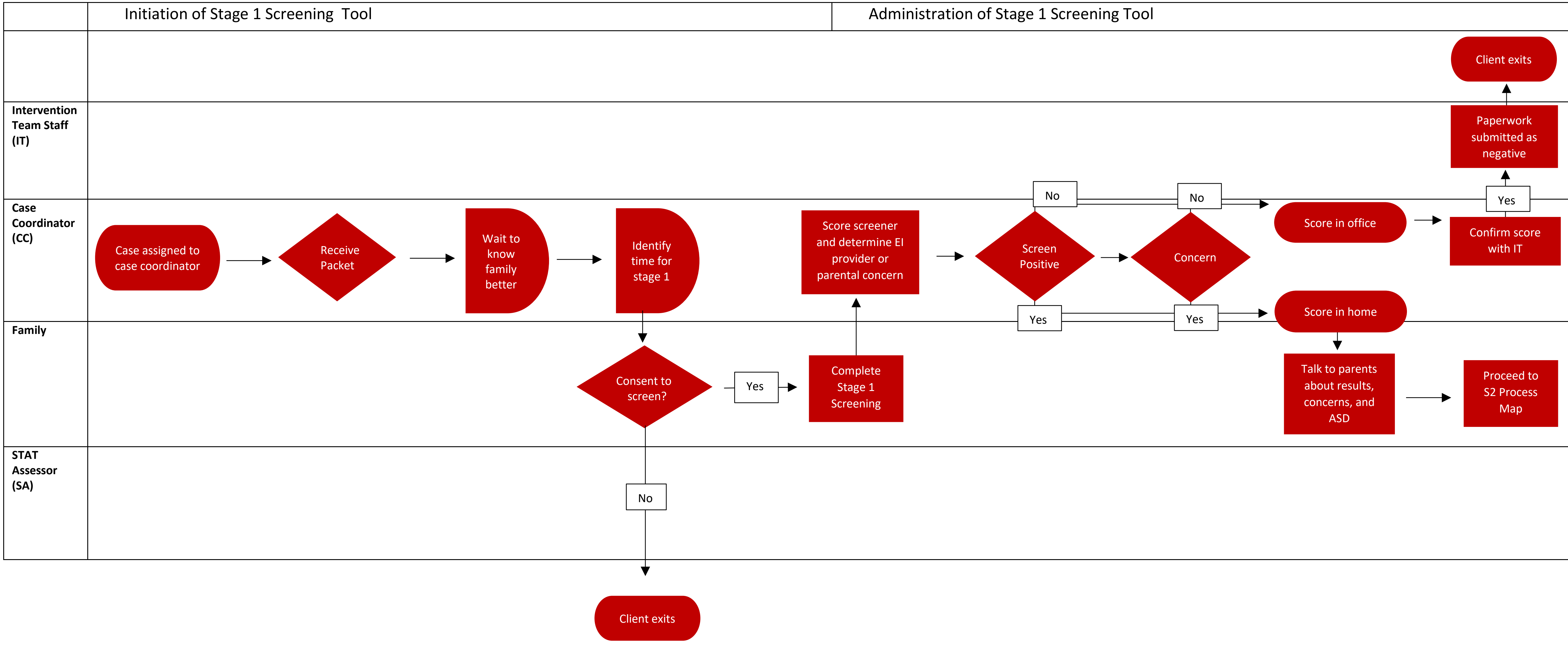

Supplement Table 1-B. Illustrative Example of Process Map "As Envisioned," Stage 2 Screening and Diagnostic Evaluation

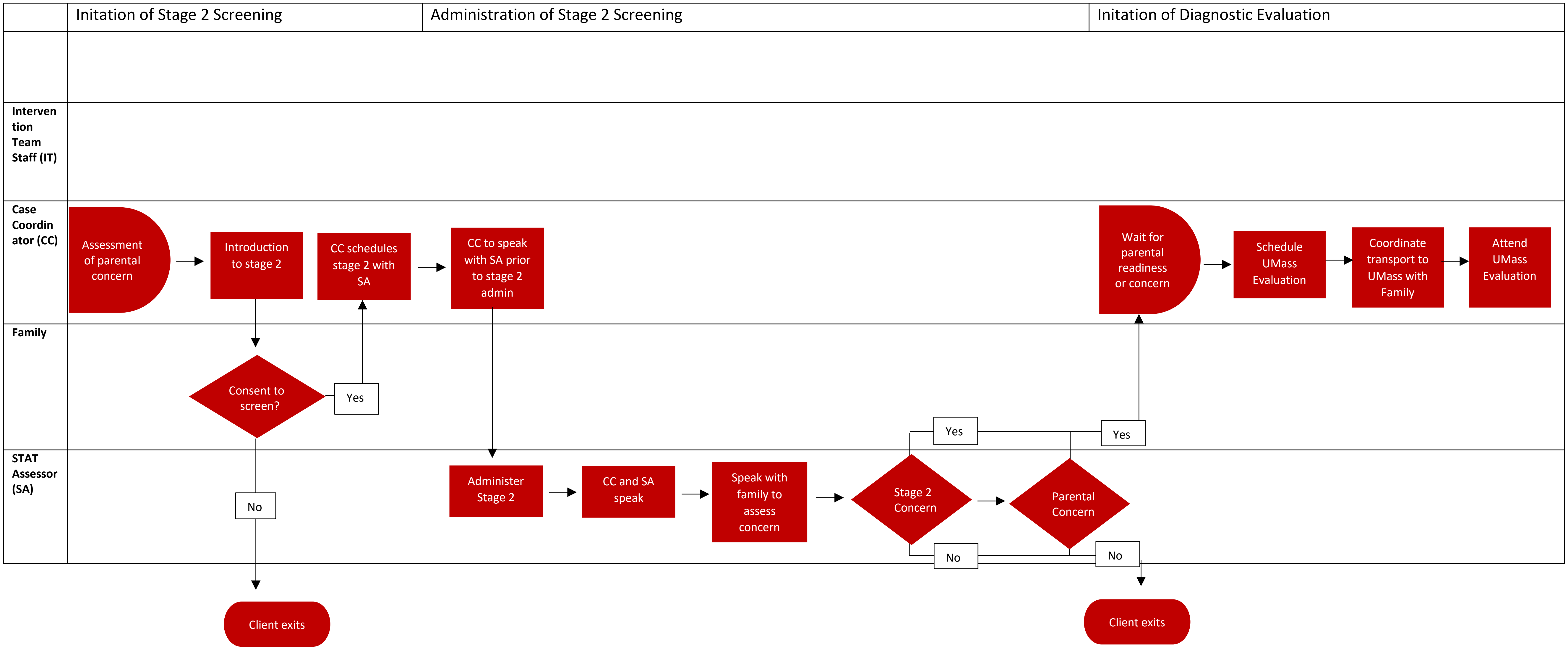

Supplement: Supplementary file 1 [file S205986612000014Xsup001.pdf]
